# Pulmonary lobar root en masse clamping and stapling technique: a quick pulmonary lobectomy

**DOI:** 10.1186/1749-8090-10-S1-A141

**Published:** 2015-12-16

**Authors:** Mitsuhiro Kamiyoshihara, Hitoshi Igai, Takashi Ibe, Natsuko Kawatani

**Affiliations:** 1 Department of General Thoracic Surgery, Maebashi Red Cross Hospital, Maebashi, Japan

## Background/Introduction

Most thoracic surgeons have experienced difficulty dissecting the pulmonary hilus because of scarring. In such potentially dangerous situations, we advocate a method of clamping and severing the pulmonary lobar root structure en masse.

## Aims/Objectives

The aim of this study was to evaluate en masse lobectomy, particularly considering the constellation of pulmonary vessels and the bronchus.

## Method

Using a stapler or vascular clamp forceps, we clamped and compressed the root of the affected lobe en masse. Care was taken to consider the remaining vessels and bronchus. We chose the direction of stapling so as to avoid overlapping the remnants of vessels and the bronchus as much as possible. When the pulmonary lobar root structure was too thick or was calcified, we clamped the entire lobar root using a vascular clamp and transfixed it with horizontal mattress sutures. We retrospectively evaluated the surgical procedures and clinical outcomes.

## Results

Twenty patients (median age, 68 years) underwent en masse lobectomy. Ten patients had benign lung disease, 7 had malignant lung disease, and 3 had suffered chest trauma. Fourteen operations were elective and 6 were emergent. Lobectomy included the right upper lobe in 3 cases, the right middle lobe in 5, the right lower lobe in 2, the right middle-lower lobe in 2, the left upper lobe in 1, and the left lower lobe in 7. A stapler was used in 18 patients, and sutures were applied in 7 (both were used in 5 case). Morbidities included a pyothorax and a persistent air leak; both made a recovery. Mortality included one emergency case of acute respiratory distress syndrome. No bronchopleural or bronchovascular fistulas occurred.

## Discussion/Conclusion

We believe that the en masse lobectomy is an alternative and necessary technique in critical or unexpected situations, possibly as a last resort.

